# Adherence to Intranasal Steroids in Chronic Rhinosinusitis with Nasal Polyposis Prior to and during Biologic Therapy: A Neglected Matter

**DOI:** 10.3390/jcm13041066

**Published:** 2024-02-13

**Authors:** Francesca Norelli, Michele Schiappoli, Gianenrico Senna, Patrick Pinter, Bianca Olivieri, Giancarlo Ottaviano, Eugenio De Corso, Marco Caminati

**Affiliations:** 1 Department of Medicine, University of Verona, 37129 Verona, Italy; francescanore@gmail.com (F.N.); gianenrico.senna@aovr.veneto.it (G.S.); 2 Allergy Unit and Asthma Center, Verona Integrated University Hospital, 35134 Verona, Italy; michele.schiappoli@aovr.veneto.it (M.S.); biancaolivieri92@gmail.com (B.O.); 3 Division of Otorhinolaryngology, Department of Surgery, Dentistry, Gynecology, and Pediatrics, Verona University Hospital, University of Verona, 37129 Verona, Italy; patrick.pinter@aovr.veneto.it; 4 Otolaryngology Section, Department of Neurosciences, University of Padova, 35122 Padova, Italy; giancarlo.ottaviano@unipd.it; 5 Otorhinolaryngology Unit, A. Gemelli University Hospital Foundation IRCCS, 00168 Rome, Italy; eugenio.decorso@policlinicogemelli.it

**Keywords:** adherence, nasal polyposis, chronic rhinosinusitis, intranasal corticosteroids

## Abstract

Adherence to treatment is essential in chronic rhinosinusitis with nasal polyposis (CRSwNP). Intranasal corticosteroids (INCS) are the first-line therapy, followed by systemic corticosteroids and surgery if needed. In cases of refractory disease, biologics are added to conventional treatment, making adherence to INCS crucial in assessing eligibility for these targeted therapies. The purpose of this review is to examine INCS adherence assessment and rate, before starting and during biologic therapy. We conducted a comprehensive literature review focusing on INCS adherence in CRSwNP treated with biologics, including randomized controlled trials and real-life studies. The search extended to studies on allergic and non-allergic rhinitis to provide broader insights into tools to assess the INCS adherence. The result was that adherence to INCS in CRSwNP is underexplored, with only a few studies addressing it directly. Various tools for adherence assessment have been identified, but none are universally accepted as standard. The review also highlights the complexity of factors influencing adherence rates. Effective CRSwNP management requires a paradigm shift to prioritize adherence in treatment guidelines and clinical practice. The review advocates for improved adherence assessment tools, a deeper understanding of influencing factors, and the integration of personalized medicine approaches, especially for biologic therapies.

## 1. Introduction

The WHO defines adherence as “the extent to which the persons’ behaviour (including medication-taking) corresponds with agreed recommendations from a healthcare provider” [1]. Adherence to medication is a crucial part of patient care and required for reaching clinical goals; it represents a critical issue in any chronic condition, even more in respiratory diseases. In the case of bronchial asthma and COPD, according to literature evidence, only 20–30% of patients regularly take the prescribed medication [2] and treatment underuse has been commonly described in both adults and children [3,4,5]. Furthermore, when talking about severe asthma, a low adherence has been reported both before starting and during biologic therapy [6], although poor disease control and an increased risk of asthma exacerbation have been described as the consequence of inappropriate asthma management [7,8]. CRSwNP represents the most relevant comorbidity affecting severe asthma patients in terms of prevalence and the burden of the disease and it shares with asthma an eosinophilic inflammatory background as the major pathobiological driver [9,10]. Its prevalence in the general population is estimated to be around 3–4% with a variability based on geographical area, but it increases to 40–60% in patients suffering from severe eosinophilic asthma [10,11]. Though this is not a life-threatening condition, it heavily impacts the quality of life of affected patients, particularly because of the nasal obstruction with concomitant hypo/anosmia and sleep disturbances it causes [12]. Interestingly, a similar scenario can be observed in this condition compared to asthma regarding the adherence to therapy, specifically about the use of intranasal corticosteroids (INCS). A regular treatment with INCS is considered, in fact, the cornerstone of CRSwNP management according to recent guidelines [13]. Despite this, in a recent survey authors demonstrated that the general perception of ENT about the adherence to INCS of CRSwNP patients is quite variable and rather low, ranging between 50 and 70% [14]. Of note, monoclonal antibodies have been approved as add-on therapies to INCS in severe cases of CRSwNP, meaning that eligibility to biologic therapy implies an unsuccessful regular use of nasal steroids in addition to the contraindicated/unsuccessful use of OCS and/or surgery. In the case of no history of previous sino-nasal surgery, four criteria are required to start biologics according to EUFOREA consensus: evidence of type 2 inflammation, comorbid asthma, significant loss of smell, significant impaired quality of life or need for systemic CS (two or more courses in the past year) [15].

The aim of this review is to explore INCS adherence assessment and rate, before starting and during biologic therapy, in randomized controlled trials (RCTs) and in real-life studies investigating the efficacy of biologic drugs for CRSwNP. Furthermore, we reviewed the main tools used in different studies to evaluate adherence to INCS in both CRSwNP and allergic rhinitis, also assessing the factors related to better and worse adherence rates.

## 2. Treatment Options and Adherence in Current Guidelines

The management of CRSwNP is often challenging. According to the current guidelines, the cornerstone of the management of CRSwNP consists of treatment with intranasal corticosteroids due to their strong and multidirectional anti-inflammatory activity. There are different corticosteroid delivery options, including nasal sprays, irrigation, nebulization, a steroid-eluting stent and direct infiltration. Nasal sprays and rinses can be found over the counter, so they are easily affordable. At the same time, the delivery of corticosteroids through nasal spray and rinses may not be sufficient to reach some areas such as the frontal sinus. When comparing corticosteroid nasal irrigation to sprays, nasal irrigation was found to be superior to nasal sprays in post-surgical patients [16].

When inadequate to achieve disease control, short courses of oral corticosteroids (OCS) can be prescribed, even if in clinical practice systemic corticosteroids are commonly used for longer timeframes due to the high frequency of nasal polyps recurrence. OCS are often used in an abusive manner, exposing the patient to significant systemic side effects. Based on an Italian survey, the most frequently observed adverse events related to the administration of systemic steroids are hypertension (57.6%), hyper-glycaemia (55.76%), insomnia (50%), anxiety (23.27%), diabetes (23.04%), mood changes (21.66%), increased appetite (12.67%) and glaucoma (10.83%) [14]. Patients sometimes describe the adverse effects related to the use of OCS as even more disabling than the CRSwNP symptoms themselves [17]. The International Consensus Statement on Allergy and Rhinology: Rhinosinusitis gives a grade A recommendation for the use of short-term OCS in the management of CRSwNP but does not recommend long-term use [18]. Likewise, the guidelines in the European Position Paper on Rhinosinusitis and Nasal Polyps (EPOS) give a grade A recommendation, corroborated by level 1a evidence, for the use of OCS [13].

There is a need for high-level studies to define the safest and most effective regimen and dosage of OCS. A review by Ahmed et al. including seven articles shows that the daily doses vary for prednisolone from 15 mg to 1.3 mg/kg, with total doses ranging from 150 to 440 mg. In addition, several studies use the same regimen for both CRSwNP and CRSsNP [19].

As a further step, endoscopic sinus surgery (ESS) is considered for CRSwNP patients whose condition is not adequately controlled by the medical treatment. The aim of the surgical approach is to unblock the nasal cavities and widen the ostium of the paranasal sinuses in order to restore respiratory function and allow INCS to reach the sinuses’ epithelium; however, the risk of disease recurrence cannot be effectively prevented by the surgical intervention, which does not impact the underlying inflammation [13,20]. Isaman et al. carried out a retrospective study to define the impact of functional endoscopic sinonasal surgery (FESS) in reducing OCS burden and healthcare resource utilization. They found that in real-world US practice, patients with CRSwNP undergoing FESS had a similar OCS burden to those not undergoing FESS, and with similar costs during follow-up, indicating a high treatment burden and unmet needs in both groups [21].

When standard of care fails, biologic agents represent the options in the presence of features suggestive of a type 2 inflammation. More than one biologic is currently marketed for CRSwNP, addressing different targets of the underlying immunological cascade, so that the identification of the best biologic drug for each patient implies a careful endo-phenotyping of the inflammation profile [22]. FDA-approved biological therapies for nasal polyps are omalizumab, mepolizumab and dupilumab. Currently, there are neither head-to-head trials nor guidelines available to help decide between these FDA-approved biological treatments. In addition, the high cost of biologics for the health care system, even in the light of their life-long schedule, requires careful patient selection including a detailed evaluation of previous treatments and clinical responses [11]. Importantly, most biological therapies approved for CRSwNP used intranasal corticosteroid sprays in the control arm as a standard of care [23].

In the comprehensive management of CRSwNP, adherence to nasal corticosteroid therapy is critically emphasized in the latest guidelines, and the targeted therapy is not intended to replace traditional treatment, which is recommended on a regular basis as a concomitant medication during biological treatment. The European Position Paper on Rhinosinusitis and Nasal Polyps (EPOS) 2020 [13] positions intranasal corticosteroids as the central therapeutic approach, stressing their importance for the sustained, long-term management of CRS symptoms. Notably, the guidelines favor corticosteroids such as mometasone furoate, fluticasone propionate and fluticasone furoate due to their minimal systemic absorption and lower risk of adverse effects. This choice reflects the understanding of the balance between efficacy and safety in chronic conditions.

EPOS 2020 also underlines the proven effectiveness and safety of the long-term use of nasal corticosteroids in CRSwNP. These medications have shown significant benefits in terms of improving patients’ quality of life, reducing nasal polyp size, and even preventing the recurrence of polyps when used postoperatively. This recommendation is particularly notable, even though direct evidence in the postoperative setting is somewhat lacking, indicating a strong consensus on their utility based on indirect evidence and clinical experience.

Beyond the choice of medication, EPOS provides detailed recommendations on the optimal administration of nasal corticosteroids. This includes practical tips such as priming the bottle before use, shaking the bottle to reduce the viscosity of the drug suspension, and correct techniques for spraying to ensure effective drug delivery and minimize risks such as nasal bleeding or septum irritation. Special considerations are given for patients with physical limitations or coordination challenges, highlighting the need for individualized patient education and support.

The European Forum for Research and Education in Allergy and Airways Diseases (EUFOREA) [24] complements these recommendations by emphasizing the broader context of patient education in disease management. It underscores the role of nasal rinsing and corticosteroids as foundational in the EUFOREA management flowchart for CRSwNP, underscoring these as initial and secondary steps in treatment. This approach implicitly involves a commitment to patient adherence, recognizing that the success of the overall management plan hinges on the consistent and correct use of these baseline therapies.

Moreover, both EPOS and EUFOREA guidelines acknowledge the multifaceted nature of CRSwNP management. They highlight the need for personalized approaches, considering factors like phenotyping and endotyping in surgical prognosis and postoperative care.

The ARIA-ITALY multidisciplinary consensus on nasal polyposis defines biologic drugs as a possible beneficial integration or improvement of the standard therapy [25]. It suggests starting treatment with a biologic drug in case of relapse after surgery despite therapy with nasal steroids.

Guidelines always consider biologic drugs as an add-on therapy, so standard-of-care therapy consisting of intranasal saline irrigations and INCS should be continued. At present, the length of time of treatment with biologics has not been well defined, and there are no indications about reducing the dose of INCS over time.

In summary, the guidelines provide a robust and detailed framework for the use of nasal corticosteroids in CRSwNP. They emphasize not only the clinical efficacy and safety of these treatments but also the crucial roles of patient education, adherence, and personalized care in achieving optimal therapeutic outcomes in managing this complex and chronic condition.

## 3. Evaluation of Adherence to INCS in RCTs and Real-Life Studies on Biologics in CRSwNP

We conducted a comprehensive PubMed search of full-length articles in English using the following key terms: “chronic rhinosinusitis with nasal polyposis”, ‘’adherence to intranasal corticosteroids’’ or “biologic therapy in chronic rhinosinusitis with nasal polyposis”. Selected articles included randomized controlled trials and real-life studies about patients with CRSwNP receiving therapy with biologics prescribed according to current guidelines. Papers that were judged more informative for the purpose of the review were retained (17 articles) (Table 1) [26,27,28,29,30,31,32,33,34,35,36,37,38,39,40,41,42]. We excluded reviews and network meta-analysis (188 articles), case reports and case series (6 articles).

Although local corticosteroids are considered to be the first-line therapy in CRSwNP, adherence to INCS is rarely explored or even mentioned in both randomized controlled trials (RCTs) and real-life studies investigating the efficacy of biologic drugs for CRSwNP.

Surprisingly, only three of the reviewed studies clearly mention assessing adherence to INCS before and/or during biologic therapy [29,33,41]. De Corso et al. defined patients taking INCS < 5 days/week as non-adherent, whilst the full intake of the prescribed treatment was requested in order to be classified as adherent. At study baseline, the adherence rate to INCS was overall 85% and it slightly increased over the biologic therapy time [29]. A different definition was provided by Pecorari and co-authors, who considered adherence to INCS therapy as the regular daily administration for at least 50% of the prescribed timeframe [41]. Bachert et al. estimated adherence according to the data stored by an electronic diary assigned to the patients and recording mometasone furoate nasal spray daily use [33]. Another randomized, placebo-controlled trial by Bachert et al. investigating the efficacy and safety of benralizumab in CRSwNP claims that they provided patients with mometasone furoate nasal spray to ensure the consistency of background INCS during the 5-weeks run-in period and throughout the study. Yet, they do not clarify if adherence was assessed, for example by keeping count of the provided sprays [42]. The remaining studies we considered did not clearly mention assessing adherence to INCS, despite the ineffective use of INCS being a criterion for biologic drug prescription.

## 4. Tools for Adherence Assessment and Factors Affecting Adherence Rate

Several tools are available to evaluate adherence to INCS, but none of them can be considered as the gold standard. To investigate the most commonly used tools in clinical practice to assess adherence to INCS in both adults and children, we selected a series of real-life studies about CRSwNP, allergic rhinitis and non-allergic rhinitis (Table 2) [43,44,45,46,47,48,49,50,51,52,53]. We considered allergic and non-allergic rhinitis as well to achieve additional insights and methodologies, since these nasal pathological conditions all share a therapeutic approach involving the use of INCS. We even focused on modifiable and non-modifiable factors related to better or worse adherence rates.

Keeping detailed logs of nasal spray use and recording the time and dose of each administration represents an easy way of monitoring adherence. However, this method requires strong and maintained patient collaboration and might not be reliable over time [54]. As an alternative option, patients can be asked to complete questionnaires [45,47,48,49,50,51,52,53], which may include questions about administration habits and related issues, or reasons for treatment non-compliance. Among them, the Moriski questionnaire, which is a generic, self-reported, medication-taking-behavior scale initially validated for hypertension, is widely used, although not specifically developed for nasal therapy [55]. Emre Ocak et al. used an eight-item questionnaire to assess medical adherence in allergic rhinitis in two different studies conducted on adult patients and children, respectively. In both cases, a good adherence was found. However, patient reliability is essential for that tool as well, and its best performance is limited to short-term studies [50,52].

Measuring the weight of medication consumed (WMC) seems to be a more reliable method to evaluate adherence to INCS, as it is performed directly by the investigator. Interestingly, when this kind of evaluation tool is used, a difference between the compliance reported by patients and the one detected by medication weighing stands out. C. Y. Loh et al. found out that less than 50% compliance was reported only by 1.6% of the study population by direct questioning, but then detected at 11% by WMC [43,44].

Electronic monitoring might be useful when nasal sprays are equipped with electronic devices that automatically record the timing of each administration. Those devices can be connected to a monitoring system that allows data about nasal spray use to be collected. These electronic tools provide the unique opportunity of an accurate and objective record of treatment adherence, overcoming the patients’ reliability issue. Data collected can be used to define patterns of nasal spray administration and identify any adherence issues. For example, if a patient frequently forgets to use the nasal spray during daylight hours, it may be necessary to provide reminders. So far, a major limitation of electronic monitoring is related to the restricted number of nasal devices adequately equipped. Even in that case, patient cooperation is essential and the low familiarity with electronic devices and perhaps costs may constitute a barrier for their widespread use [56].

Furthermore, some of these studies have highlighted a few factors related to better or worse adherence rates. Lack of symptom improvement, fear of steroid side effects and prior negative experience represent the main barriers towards adherence. Even having more than two dependent children and smoking habits have been described as factors related to lower adherence rate [50,52]. On the other hand, P. Singh et al. evidenced that medication adherence in adult patients with allergic rhinitis was significantly superior in patients with elevated total IgE, house dust mite sensitization and severe total nasal symptom score (TNSS). In fact, adherence was 2 times more likely in Dermatophagoides pteronyssinus allergy, 2.7 times more likely in elevated total IgE and 15 times more likely in severe TNSS at first visit [49]. A higher level of education has also been described as a determinant of high adherence rate, along with female sex and age between 28 and 59 years old [45,52]. Furthermore, receiving daily reminders as SMS or app notifications on one’s cell phone has a significant role in increasing adherence [46,47]. Feng et al. demonstrated that using a mobile service providing daily reminders such as text messages, images and videos about updated knowledge about CRS, the importance of INCS and correct spray technique improved adherence up to 93.9% [46]. In patients suffering from CRS, even a history of previous sinus surgery and comorbid asthma increase the adherence rate to INCS [51]. Administration technique is another critical issue which may negatively affect adherence rate. In fact, the use of the homo-lateral hand for delivering the intranasal steroid can be responsible for irritation and epistaxis in 20% of patients [57]. However, the inhalation technique is rarely assessed in real life. Finally, the cost of nasal treatment, if paid out of pocket by the patients, may further impact adherence rate [48].

## 5. Discussion

Treatment adherence is crucial for the achievement of the full therapeutic effect of any treatment. Its relevance is also part of the clinical assessment when defining a relapsing/refractory condition despite the ongoing therapy, which must be properly taken to avoid misclassifications and misdiagnosis. In addition, some therapeutic options, including biologic drugs, are intended as “add on” treatments, which again implies an optimal adherence rate before and after the introduction of the targeted therapy [13].

It is the case that monoclonal antibodies currently marketed for CRSwNP, which are recommended in difficult-to-treat patients who are not achieving disease control with traditional treatment. Although the existence of peculiar inflammatory phenotypes poorly responding to steroid topical treatment has a biologic plausibility, no specific markers allow us to detect it, so that the proper intake of topical treatment remains crucial for the evaluation of eligibility to biologic therapy [42]. Furthermore, the efficacy of targeted treatment itself might be hampered by an unsatisfactory adherence to topical treatment.

A significant finding of this review is the apparent neglect of adherence assessments in the studies on CRSwNP. While various tools to measure adherence are available, none are fully valid, reliable, or sensitive. The studies reviewed reveal a fragmented and inconsistent approach to adherence assessment, with methods varying significantly between studies. In fact, while different tools are available to explore patients’ adherence, none of them are completely valid, reliable and sensitive, and a combination of methods is recommended in order to integrate and validate the information coming from patients’ reports with objective measures [58]. This makes investigating the adherence rate undoubtedly complex and time-consuming, which is a relevant aspect in real-life setting management. The studies included in this review have several limitations that need to be acknowledged. Firstly, the number of studies specifically focusing on adherence in CRSwNP is limited. This gap in the literature restricts the depth of analysis and conclusions that can be drawn specifically for CRSwNP patients. Secondly, the varied tools used to assess adherence across these studies make it challenging to compare and synthesize findings effectively. The disparity in methodologies ranges from self-reported questionnaires to more objective measures like electronic monitoring, each with its own set of limitations in accuracy and reliability. Additionally, some of the studies reviewed do not exclusively focus on CRSwNP but include conditions like allergic or non-allergic rhinitis. While these studies are relevant due to the shared treatment approach, the differences in disease features and patient populations mean that findings may not be entirely applicable to CRSwNP.

Despite these limitations, the review highlights a clear need for a more standardized and systematic approach to adherence assessment in CRSwNP.

In fact, confirming the regular INCS intake before and during targeted therapy prescription is a matter of both precision medicine and sustainability. For that reason, at least the use of the simplest tool, although not fully accurate in exploring the adherence rate, should be widely implemented and considered a mandatory requirement for the biologic treatment prescribers.

Learning from approaches in the management of other related diseases, such as asthma, the adoption of specific tools to assess adherence could be a promising strategy for CRSwNP. For example, the “Test of Adherence to Inhalers” (TAI) is a validated 12-item questionnaire designed to assess adherence to inhaler therapy in asthma and COPD patients. Its effectiveness has been proven through a study involving over a thousand patients, demonstrating its reliability in identifying non-adherence and categorizing barriers to inhaler use. Applying a similar approach to intranasal corticosteroids in CRSwNP could be beneficial, offering a targeted tool to evaluate and enhance adherence in this patient group [59].

## 6. Conclusions

This comprehensive review highlights the pivotal yet often underestimated role of adherence in the management of CRSwNP, especially concerning the use of INCS. Despite being a critical component in the management of CRSwNP, as outlined in clinical guidelines, adherence is frequently assumed rather than actively ensured in clinical practice. This oversight can lead to significant disparities between prescribed treatment plans and patient adherence, ultimately affecting the efficacy of disease management and patient outcomes.

The review has shown that, while clinical guidelines do address the importance of adherence, there is a noticeable gap in how this is translated into real-world clinical settings. There is a critical gap also in the literature, where adherence to INCS in the context of biologic therapy for CRSwNP is insufficiently assessed or reported.

This review underscores the need for a more nuanced understanding of the factors influencing adherence. It is clear that patient behavior, beliefs, and the perceived burden of treatment play significant roles in adherence rates. The complexity of the disease, the chronic nature of the treatment, and the potential side effects of medications such as corticosteroids and biologics can significantly impact patient willingness and the ability to adhere to treatment protocols. Additionally, the role of healthcare providers in supporting adherence, through patient education, clear communication, and regular follow-up, cannot be overstated.

## 7. Future Directions

There is an urgent need to prioritize and improve adherence assessments in CRSwNP management. As highlighted by the reviewed articles in Table 2, there are different well-known factors related to lower adherence rate, such as prior negative experiences, bothersome effects, low education level, smoking habit and having more than two dependent children. In clinical practice, the early identification of those factors should be part of the patient assessment, as adherence-related “treatable traits” to be specifically addressed prior to treatment prescription. In particular, negative experiences or side effects with previously prescribed drugs deserve a deeper level of sharing with the patient about the treatment rationale and outcomes. Similarly, low-educational-level patients deserve a more dedicated explanation about the therapeutic plan. Also, the baseline identification of the determinants mentioned above suggests to the clinician a stricter monitoring of therapy adherence. As to date a specific standardized and validated tool to assess adherence is not available yet, questionnaires like MMAS-8, filled out directly by patients, provide a quick and easy method in clinical practice, so they should be applied regularly. Of course, measuring a canister’s weight at every visit enables the clinician to know how much medication the patient has actually used, so it provides more reliable data and should be performed when possible.

When available, especially in patients with a high risk of low adherence, a tech tool able to send daily reminders and information about the disease and the importance of INCS use should be definitely used as it significantly improves the adherence rate.

Future studies should focus on developing and validating more effective tools for measuring adherence, integrating patient-reported data with objective measures to provide a comprehensive view of treatment compliance. This approach would not only enhance the accuracy of adherence assessment, but also contribute to a deeper understanding of the factors influencing patient behavior.

Additionally, there is a call for personalized medicine approaches in the treatment of CRSwNP. This includes refining phenotyping and endotyping methods to better tailor treatments to individual patient profiles, particularly when considering biologic therapies. As we advance, the focus should also be on the broader aspects of patient care, such as education, support systems, and technological interventions like reminder systems, which have shown promise in improving adherence rates.

In the realm of biologics, there is a need for continued research into identifying biomarkers that can more accurately predict responses to these therapies. This would aid in the more precise selection of patients for biologic treatment and ensure the sustainability and cost-effectiveness of these innovative therapies. Moreover, the integration of adherence monitoring into routine clinical practice should be emphasized, especially for patients being considered for biologic therapies, to ensure that these useful medications are used optimally and effectively.

In conclusion, enhancing adherence to INCS and biologics in CRSwNP requires a multifaceted approach, involving improved assessment tools, patient education, personalized treatment strategies and a better understanding of the barriers to adherence. As the field progresses, these efforts will be crucial in optimizing treatment outcomes and improving the quality of life for patients with CRSwNP.

## Figures and Tables

**Table 1:** Assessment of adherence to INCS in randomized controlled trials and real-life studies investigating the use of biologics in CRSwNP.

Authors	Year of Publication	Biologic	Study Design	Patients	Study Duration	INCS Use	Adherence to INCS
Philippe Gevaert et al. [26]	2020	Omalizumab	Two replicate randomised, multicenter, double-blind, placebo controlled phase 3 trials	POLYP 1 *n* = 138 POLYP 2 *n* = 127	24 weeks	≥4 weeks of INCS therapy before screening Mometasone furoate 100 mcg into each nostril twice a day or once daily during the 5-week run-in period and throughout the trial	Not assessed
Joseph K Han et al. [27]	2021	Mepolizumab	Randomised, double-blind, placebo-controlled, parallel-group, phase 3 trial	*n* = 407	52 weeks	Mometasone furoate 100 mcg into each nostril twice a day for ≥8 weeks before screening and throughout the study	Not assessed
Claus Bacher et al. [28]	2019	Dupilumab	Two multicentre, randomised, double-blind, placebo-controlled, parallel-group phase 3 trials	SINUS-24 *n* = 276 SINUS-52 *n* = 448	SINUS-24 = 24 weeks SINUS-52 = 52 weeks	Mometasone furoate 100 mcg into each nostril twice a day during the 4-week run-in period and throughout the trial	Not assessed
Eugenio De Corso et al. [29]	2023	Dupilumab	Multicentric, retrospective, observational phase IV real-life study	*n* = 648	52 weeks	INCS before and throughout the study (mometasone or budesonide or other)	Assessed before and throughout the study
Somaira Nowsheen et al. [30]	2021	Dupilumab	Retrospective cohort study	*n* = 29	11 months	Previous treatment with INCS in all patients Concurrent INCS in 65.5% of patients	Not assessed
Eva C. Meier et al. [31]	2021	Omalizumab, mepolizumab or benralizumab	Real-life, retrospective study	*n* = 29	Different for each treatment regimen	INCS throughout the study	Not assessed
Rik Johannes Leonardus van der Lans et al. [32]	2021	Dupilumab	Real-life, prospective, observational study	*n* = 98 *n* = 26	24 weeks 48 weeks	Not assessed	Not assessed
Claus Bachert et al. [33]	2016	Dupilumab	Randomised, double-blind, phase 2, placebo-controlled, parallel-group study	*n* = 60	16 weeks	INCS treatment for at least 8 weeks before screening Mometasone furoate 100 mcg into each nostril twice daily during the 4-week run-in period Mometasone furoate 200 mcg twice a day or once daily throughout the study	Assessed throughout the study
Corrado Pelaia et al. [34]	2021	Dupilumab	Real-life, single-center, observational study	*n* = 20	4 weeks	Not assessed	Not assessed
Elena Cantone et al. [35]	2022	Dupilumab	Real-life, retrospective, observational study	*n* = 53	24 weeks	INCS before the study Mometasone furoate 100 mcg in each nostril once daily throughout the study	Not assessed
Giancarlo Ottaviano et al. [36]	2022	Dupilumab	Real-life, observational study	*n* = 47	12 months	INCS throughout the study	Not assessed
Sara Torretta et al. [37]	2022	Dupilumab	Real-life, observational study	*n* = 80	12 months	INCS throughout the study	Not assessed
Florian Jansen et al. [38]	2022	Dupilumab	Real life, single-centered, retrospective single-arm longitudinal study	*n* = 40	13 months	Continuous usage of INCS in the maximum dosage before starting dupilumab	Not assessed
Cosimo Galletti et al. [39]	2023	Dupilumab	Monocentric, real-life, observational cohort study	*n* = 66	12 months	INCS in the preceding two years before starting dupilumab	Not assessed
Eustachio Nettis et al. [40]	2022	Dupilumab	Real-life, multicentric, observational, prospective study	*n* = 82	16 weeks	Not assessed	Not assessed
Giancarlo Pecorari et al. [41]	2023	Dupilumab	Real-life, observational study	*n* = 52	12 months for 35 patients <12 months for 13 patients	Mometasone furoate 100 μg into each nostril twice a day throughout the study	Assessed before and throughout the study
Claus Bachert et al. [42]	2022	Benralizumab	Randomised, double blind, placebo controlled trial	*n* = 413	40 weeks	Study-provided mometasone furoate nasal spray (400 mcg total daily dose) during 5-week run-in period and throughout the study	Not explicitly mentioned

**Table 2:** Different tools used to assess adherence to INCS among studies in CRSwNP and allergic or non allergic patients.

Authors	Disease	Patients	Adherence Evaluation Tool	Adherence Percentage	Factors Related to High Adherence	Factors Related to Low Adherence
A. Pizzulli et al. [43]	Allergic rhinitis	*n* = 70 (children and adolescents)	Measuring canister’s weight	48.4% in the group undergoing telemonitoring	Internet-based telemonitoring system	Not assessed
C. Y. Loh et al. [44]	Allergic and non-allergic rhinitis	*n* = 63	Direct questioning and measuring the weight of medication consumed (WMC)	77.8% reported a forgetfulness of using medication for 1–5 times Less than 50% compliance was reported by 1.6% but detected in 11% by WMC	Not assessed	Not assessed
Almutairi TA et al. [45]	Allergic rhinitis	*n* = 375	Questionnaire based surveys	71.5%	High education level	Male sex Safety concerns Age < 28 y.o. Age > 59 y.o. Smoking habit Low and middle socioeconomic status
Shaoyan Feng et al. [46]	CRSwNP	*n* = 29	Calculating percentage of the medicine actually taken by the patients based on the medicine dose-count on the returned device (bottle of Rhinocort Aqua)	93.9% in the WeChat group 76.6% in the control group	Daily WeChat services on cell phone providing text messages, images and even videos about updated knowledge about CRS, importance of INCS and correct spray technique	Not assessed
Kuiji Wang et al. [47]	Allergic rhinitis	*n* = 50	Self-reported adherence	60% in the SMS group 28% in the control group	Daily SMS reminder	Not assessed
Meha G. Fox et al. [48]	Allergic rhinitis	*n* = 32	Semi-structured interview	Not assessed	Creating memory triggers	Prior negative experience Safety concerns
Prempreet Kaur Manjit Singh et al. [49]	Allergic rhinitis	*n* = 185	Patients’ diaries	59.5%	Elevated total IgE House dust mite allergy Severe TNSS* at the first visit	Safety concerns Bothersome effects
Emre Ocak et al. [50]	Allergic rhinitis	*n* = 76 children	Morisky medical adherence scale (MMAS-8)	71.51%	Not assessed	More than 2 dependent children to the caregiver
Katie M. Phillips et al. [51]	CRS	*n* = 174	Self-reported adherence	44.3% at the time of presentation 60.3% at follow up visit	History of previous sinus surgery Aeroallergen hypersensitivity Comorbid asthma Change in SNOT-22 score	Not assessed
Emre Ocak et al. [52]	Allergic rhinitis	*n* = 59	MMAS-8	mean overall MMAS-8 score 3.64 (meaning high adherence)	High education level Few side effects	More than 2 dependent children No benefit from the medication
V. Ganesh et al. [53]	Allergic rhinitis and CRS	*n* = 103	Questionnaire based surveys	71%	Not assessed	Lack of symptom improvement Side effects

## Data Availability

The original contributions presented in the study are included in the article, further inquiries can be directed to the corresponding author.
